# Proximal Gastrectomy versus Total Gastrectomy for Siewert Type II Adenocarcinoma of the Esophagogastric Junction: A Comprehensive Analysis of Data from the SEER Registry

**DOI:** 10.1155/2019/9637972

**Published:** 2019-12-31

**Authors:** Kaixuan Zhu, Yingying Xu, Jiaxin Fu, Farah Abdidahir Mohamud, Zongkui Duan, Siyuan Tan, Zekun Zhao, Ping Chen, Liang Zong

**Affiliations:** ^1^Department of General Surgery, Northern Jiangsu People's Hospital, Clinical Medical College, Yangzhou University, Jiangsu Province, China; ^2^Department of General Surgery, Yizheng People's Hospital, Clinical Medical College, Yangzhou University, Jiangsu Province, China; ^3^Department of General Surgery, Tongji Hospital, Medical School of Tongji University, Shanghai, China

## Abstract

**Background:**

To determine the ideal surgical approach (total gastrectomy (TG) vs. proximal gastrectomy (PG)) for Siewert type II adenocarcinoma of the esophagogastric junction (AEG), we searched and analyzed the Surveillance, Epidemiology, and End Results (SEER) data.

**Methods:**

Patients with Siewert type II AEG treated by TG or PG were identified from the 2004–2014 SEER dataset. We obtained the patients' overall survival (OS) and cancer-specific survival (CSS) and stratified the patients by surgical approach. We performed a propensity score 1 : 1 matching (PSM) analysis and a univariate and multivariate Cox proportional hazards model.

**Results:**

A total of 2,217 patients with 6th AJCC stage IA–IIIB Siewert type II AEG was examined: 1,584 patients (71.4%) underwent PG, and 633 patients (28.6%) underwent TG. The follow-up time was 1–131 months. OS favored total gastrectomy before the PSM analysis (*χ*^2^ = 3.952, *p* = 0.047), but after this analysis, there was no significant difference between TG and PG (*χ*^2^ = 2.227, *p* = 0.136). The univariate and multivariate analyses identified age as an independent factor, and an X-tail analysis revealed 70 years as a cut-off point. The patients aged ≥ 70 years obtained a significant long-term OS benefit from PG compared to TG (*χ*^2^ = 8.245, *p* = 0.004), and those aged < 70 years showed no difference between TG and PG (*χ*^2^ = 0.167, *p* = 0.682).

**Conclusions:**

PG showed an equivalent survival benefit to TG in both the early and locally advanced stages of Siewert type II AEG. For elderly patients, PG is strongly recommended because of its clearer OS benefit compared to TG.

## 1. Introduction

The incidence of adenocarcinoma of the esophagogastric junction (AEG) has rapidly increased worldwide in the past two decades, and the 5-year overall survival (OS) for advanced AEG is still very poor [1–4]. Based on the anatomic relationship between the location of the tumor's epicenter and the esophagogastric junction (ECJ), Siewert et al. classified AEG into three subgroups: Siewert types I, II, and III [5]. Siewert types I and III AEG are described as very similar to esophageal cancer and gastric cancer, respectively, because of their consistence in pathology. The optimal treatment strategy for Siewert type II AEG is a matter of controversy because it is too difficult to define its origin as gastric cancer or esophageal cancer.

Initially, transhiatal, transthoracic, or transthoracoabdominal esophagogastrectomies were all acceptable approaches for Siewert type II AEG cases [6, 7]. Siewert et al. [5] thought that most type II AEG tumors are closer to proximal gastric cancer than distal esophageal adenocarcinoma, and they demonstrated that there was no significant difference between the effectiveness of extended gastrectomy and esophagectomy. In addition, the Japan Clinical Oncology Group (JCOG) 9502 randomized controlled trial (RCT) conducted in Japan showed that the left thoracoabdominal approach could not be justified to treat esophagogastric junction tumors because of this approach's increased morbidity and mortality [8]. Based on these findings, the abdominal-transhiatal approach might be an appropriate option for Siewert type II AEG cases.

A further concern was raised regarding the selection of either a proximal gastrectomy (PG) or total gastrectomy (TG) in patients with Siewert type II AEG. A retrospective study by Yamashita et al. [9] showed that lymph node metastasis in Siewert type II AEG patients rarely occurred in the no. 4, no. 5, and no. 6 lymph nodes regardless of whether the tumor epicenter was closer to the esophagus or the stomach. This finding suggested that a proximal gastrectomy might provide the same oncologic outcomes as those obtained with a total gastrectomy.

Here, to identify the optimal surgical approach (proximal vs. total gastrectomy) for patients with Siewert type II AEG, we searched and analyzed the data of the Surveillance, Epidemiology, and End Results (SEER) registry.

## 2. Patients and Methods

### 2.1. Data Source

We identified the AEG cases from the case listing section of the SEER database by using the SEER^∗^Stat 8.3.5 program (http://seer.cancer.gov). The SEER registry includes demographic information, cancer incidence data, treatment descriptions, and survival data collected from 18 population-based cancer registries, which covers approx. 28% of the population of the US [10]. The SEER database is an open database, and informed consent from patients is not required because the information from the database is deidentified.

### 2.2. Study Population

Patients were chosen specifically from the up-to-date version of the SEER database with additional treatment fields (SEER 18, 1973–2014 varying), which was based on the November 2016 submission and was released in March 2018. Although the SEER database does not use the Siewert subtypes to classify EGJ cancer, we were able to identify Siewert type II cancers specifically. Cancer that satisfies two conditions (a collaborative staging (CS) Schema V0204+entry of “EsophagusGEJunction” and a primary site entry of “Cardia, NOS”) is classified as Siewert type II cancer [11]. We consulted with SEER personnel and confirmed that this classification method is reasonable.

We collected the information of the patients diagnosed with AEG from the SEER database during the period 2004–2014, because we used the information from the CS (2004+) and the TNM (tumor, node, metastasis) American Joint Committee on Cancer (AJCC) 6th (2004) edition for the present analyses (pathological staging system). The definitions of AEG subtypes used by the *American Joint Committee on Cancer (AJCC) Cancer Staging Manual* (7th edition, 2010) and those of the AJCC 6th edition (2004) are not the same; it is not possible to move the AJCC 6th edition cases to the 7th edition completely in the SEER database. The choice of histology coding was in accordance with the *International Classification of Diseases for Oncology* 3rd edition (ICD-O-3), and the codes for the subtypes of EGJ adenocarcinoma were 8140–8147, 8160–8162, 8180–8221, 8250–8507, 8514–8551, 8571–8574, 8576, and 8940–8941. The tumor site and the tumor morphology were both part of the CS Schema v0204+entry of “EsophagusGEJunction” and primary site-labeled entry of “C16.0-Cardia, NOS”.

We restricted eligibility to the patients who were ≥18 years old for whom surgery had been performed and whose first malignant tumor primary indicator results were “yes.” The patients who had distant metastasis (M1) or AJCC stage T4 or TNM stage IV were excluded because the prognosis of tumor invasion to different organs varies greatly. We removed cases with incomplete information (e.g., unknown grade, ethnicity, or stage or no regional nodes extracted). We converted the CS tumor size codes 991, 992, 993, 994, and 995 into 5 mm, 15 mm, 25 mm, 35 mm, and 45 mm, respectively, and excluded codes 990, 998, and 999. In order to distinguish the different surgical methods, the RX Summ-Surg Prim Site was restricted to “33, 40-42, 51-52.”

We divided the surgery procedures into two groups: proximal gastrectomy (PG) and total gastrectomy (TG). The PG group was comprised of the patients treated with a partial, hemi-, or subtotal proximal gastrectomy (Surgery encode 33, 51). The TG group was comprised of the patients treated with a near-total or total gastrectomy (Surgery encode 40-42, 52). In the surgical procedure used for the patients coded as 51 or 52, only portions of the esophagus were removed, without other organs. We defined the survival period as the length of time from the patient's diagnosis to death, and the study cut-off date and last contact date were both December 31, 2014.

### 2.3. Statistical Analyses

We used the *χ*^2^ test to evaluate the differences in patient characteristics between the PG and TG groups. The Wilcoxon rank sum test was applied to determine whether there was a significant difference in tumor size between the PG and TG groups. Propensity score 1 : 1 matching (PSM) and an algorithm with a caliper of 0.05 were used to pair the PG group and TG group. The matched factors used for the PSM analysis were the follow-up period, ethnicity, AJCC stage, T stage, N stage, differentiation, and chemotherapy.

We performed Kaplan-Meier curve and log-rank tests to evaluate the differences in the overall survival (OS) and the cancer-specific survival (CSS) between the PG and TG groups before and after the PSM analysis. We applied a univariate and multivariate Cox model and the Wald test to determine the hazard ratios (HRs) and 95% confidence intervals (CIs) in adjusted data. To further test the prognostic consistency, we analyzed patient subgroups based on different patient characteristics by a Cox proportional hazards model separately between the PG group and the TG group. The survival comparisons of these subgroups were performed using the same methods as those used in the primary analysis before and after PSM.

We used a logistic regression model to calculate the propensity scores, and the *χ*^2^ test was applied to examine the covariates balance from the PG and TG groups. The cut-off values for age in 2,217 patients with Siewert type II AEG were analyzed using the X-tile program, ver. 3.6.1 (Yale University, New Haven, CT, USA), which can identify the cut-off with the maximum *χ*^2^ log-rank value as well as the minimum *p* value of survival. In all of the statistical tests, the significance level was set at *p* < 0.05, and all statistical analyses were conducted with the software package SPSS, ver. 24.0 (IBM, Armonk, NY). Forest maps of the patient subgroups' survival were drawn using Microsoft Office Excel 2007 [12].

## 3. Results

### 3.1. The AEG Patients' Characteristics

A total of 2,217 eligible patients with Siewert type II AEG and 6th AJCC stage IA–IIIB were identified from the 2004–2014 SEER dataset, including 1,584 (71.4%) patients who underwent a PG and 633 (28.6%) patients who underwent a TG. The clinical characteristics of the two groups are summarized in Table 1. As the data in the table show, there were a predominance of males and a white ethnicity predominance for Siewert type II AEG, but the reasons for these predominances are not known. In addition, significant differences in the proportion of AJCC T stage (*p* < 0.05) and tumor size were revealed between the PG and TG groups (*p* < 0.05).

### 3.2. Tumor Size by Surgical Approach

**Figure 1:**
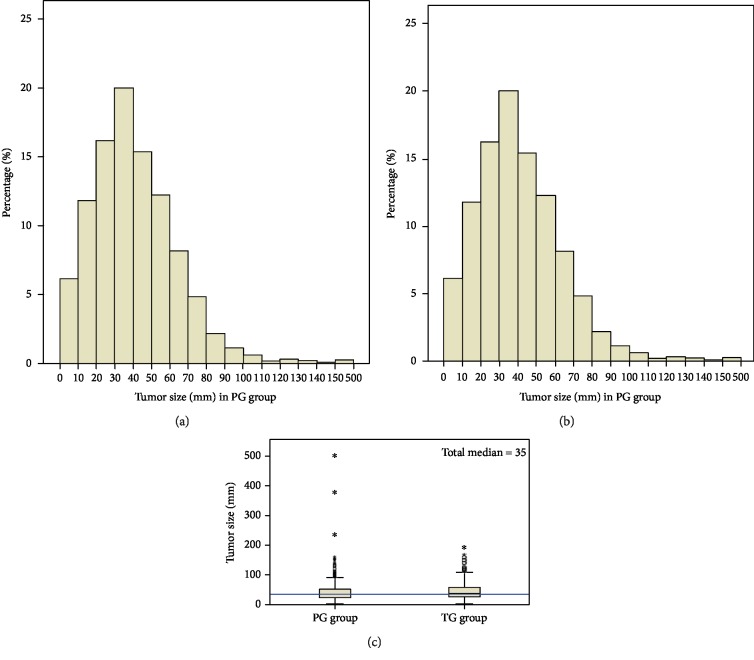
Comparison of tumor size in PG and TG groups. (a, b) Histogram of tumor sizes in the PG group and the TG group. (c) Independent sample Mann-Whitney *U* test shows the tumor sizes in the TG group were significantly larger than those in the PG group.

It cannot be ignored that the size of the AEG tumor is an important factor when selecting the surgical approach. To further examine differences in tumor size between the PG and TG groups, we used a histogram to determine the frequency distribution, and we adopted a nonparametric test to calculate the median difference (Figures 1(a) and 1(b)). With a median tumor size of 35 mm (range 1–500 mm) in all patients, the median tumor sizes in the PG and TG groups were 35 mm and 40 mm, respectively (Figure 1(c)). The independent sample was 759,277 and the Mann-Whitney *U* test Wilcoxon statistic was *Z* = 4.212 (*p* < 0.001), suggesting that there was a bias in the selection of the surgical approach. We therefore conducted a PSM analysis to decrease the effect of the selection bias. In Table 2, after the PSM analysis, a final total of 1,254 Siewert type II AEG patients were included, and the imbalance between the two groups was completely removed.

### 3.3. Survival Analysis by Surgical Approach

**Figure 2:**
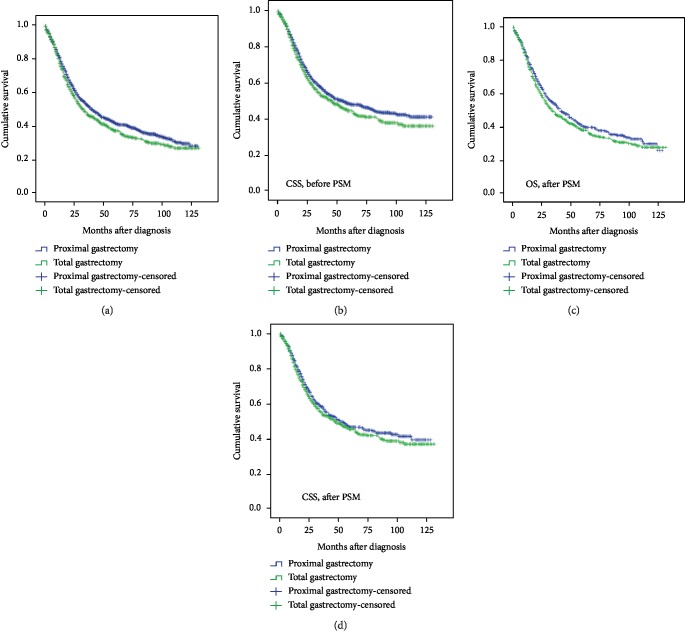
Survival comparisons in Siewert type II AEG patients between the PG group and the TG group. (a, b) OS (*χ*^2^ = 3.952, *p* = 0.047) and CSS (*χ*^2^ = 3.028, *p* = 0.073) before the PSM analysis. (c, d) OS (*χ*^2^ = 2.227, *p* = 0.136) and CSS (*χ*^2^ = 1.211, *p* = 0.271) after the PSM analysis.

To investigate whether Siewert type II AEG patients obtain a survival benefit from the surgical approach, we compared the OS and CSS between the PG and TG groups. The median OS in the PG group was 41 months, whereas in the TG group the median OS was 33 months. Before the PSM analysis, the results of our OS analysis showed that the patients who underwent a PG obtained a slight benefit compared to those who underwent TG (*χ*^2^ = 3.952, *p* = 0.047) (Figure 2(a)), while there was no significant difference in CSS between the two groups (Figure 2(b)). However, after the PSM analysis, both the OS and the CSS showed no significant difference between the PG and TG groups (Figures 2(c) and 2(d)), suggesting that a limited excision range such as that used in a PG may be sufficient for patients with Siewert type II AEG.

### 3.4. Subgroup Analyses

**Figure 3:**
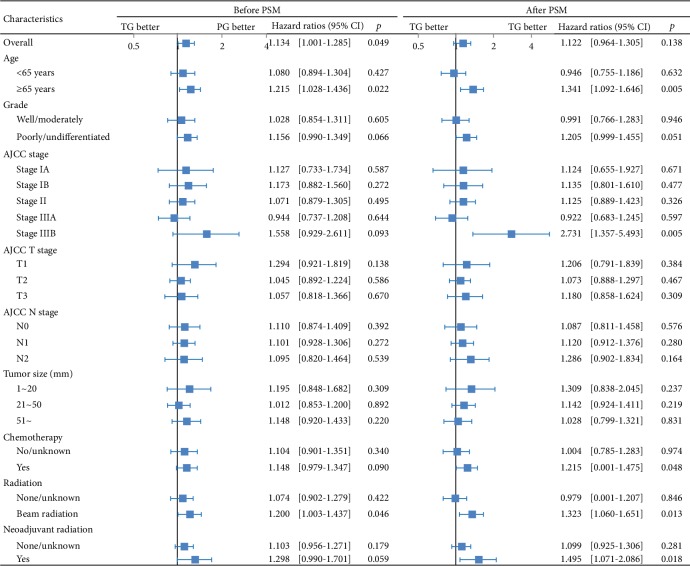
OS comparisons between PG and TG before and after the PSM in the subgroup analysis.

**Figure 4:**
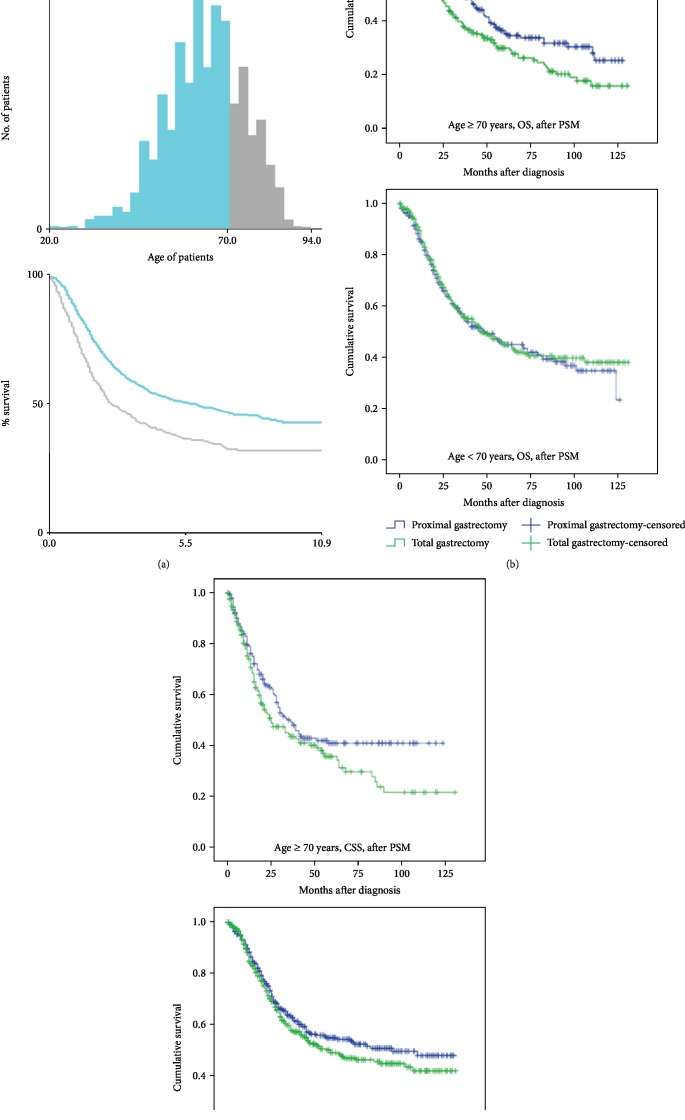
OS analysis of the age subgroups. (a) Results of the X-tile analysis of OS data of 2,217 Siewert type II AEG patients with age as a factor. The optimal cut-off value for the patients' age is shown on a histogram and Kaplan-Meier curves. The X-tail analysis showed that 70 years was the optimal cut-off value. (b) Kaplan-Meier analysis of OS between the PG and TG patients in the subgroups of patients aged < 70 years (*χ*^2^ = 0.167, *p* = 0.682) and those aged ≥ 70 years (*χ*^2^ = 8.245, *p* = 0.004). (c) Kaplan-Meier analysis of CSS analysis between the PG and TG patients in the subgroups of patients aged < 70 years (*χ*^2^ = 1.686, *p* = 0.194) and those aged ≥ 70 years (*χ*^2^ = 3.895, *p* = 0.048).

To identify independent prognostic factors for Siewert type II AEG patients, we conducted univariate and multivariate analyses. As expected, the AJCC stage, N stage, T stage, tumor size, grade, surgery approach, and the number of regional lymph nodes were identified as independent factors (Table 2). To our surprise, the factor of age played a different role in the two approaches in the subgroup analysis (Figure 3). To identify the optimum age for the selected procedure, we conducted an X-tail analysis, which revealed that 70 years was an appropriate cut-off point (Figure 4(a)). We observed that the patients ≥ 70 years old achieved a significant long-term OS benefit from PG compared to TG (*χ*^2^ = 8.245, *p* = 0.004) whereas the patients < 70 years old showed no difference in OS between TG and PG (*χ*^2^ = 0.167, *p* = 0.682) at the adjusted date, suggesting that PG should be strongly considered for elderly Siewert type II AEG patients (Figure 4(b)). Similarly, in the CSS analysis, there was a difference in survival between the younger group (*χ*^2^ = 1.686, *p* = 0.194) and the older group (*χ*^2^ = 3.895, *p* = 0.048) (Figure 4(c)).

## 4. Discussion

There is no controversy regarding the appropriate surgical approach for patients with distal esophageal (Siewert type I) cancer or proximal gastric (Siewert type III) cancer, but the treatment of patients with the true cardia adenocarcinoma (Siewert type II) continues to be a matter of debate. In general, with the principle of complete tumor removal, two surgical procedures are performed in cases of Siewert type II adenocarcinoma: extended esophagectomy and extended total gastrectomy. The oncologic outcomes of these two procedures are comparable [13, 14], and both are frequently used [9, 15]. Although the NCCN recommends that Siewert type I and type II cancers should be treated with esophageal cancer regimens and that the management of patients with type III cancer should be similar to gastric cancer regimens [16], many type II cancers are treated with a PG or TG via the transabdominal approach in clinical practice. The optimal gastric resection extent for cardia adenocarcinoma remains controversial. To determine the impact of reserving distal stomach on the survival of Siewert type II patients, we compared the TG with PG procedures in terms of their outcomes regardless of the extent and the approach of the proximal esophagus resection [17].

Some research groups have speculated that TG is superior to PG due to the increased incidence of lymph node metastasis in advanced proximal gastric cancer [18], but other evidence supports the equivalence of the two procedures in terms of survival [19–21]. In our present retrospective analysis using the SEER database, there was no significant difference in overall survival between the patients who underwent a PG and those treated with a TG after the statistical adjustment (*χ*^2^ = 2.227, *p* = 0.136). These results, in accordance with previous studies [20, 21], support the survival similarities of AEG patients treated with PG or TG, indicating that the two procedures are comparable in terms of survival outcomes.

In a 2011 study by Yamashita et al. of an AEG cohort [9], a few of the patients had parapyloric node metastasis only, and when the tumor metastasis involved multiple node stations, the nodal metastasis was mainly in the nodes of the pericardium (nos. 1, 2), lesser curve (no. 3), and the nodes along the left gastric artery (no. 7). On the other hand, a significant therapeutic benefit was obtained when the pericardial and lesser curve lymph nodes were removed, whereas the dissection of downward perigastric nodes (e.g., the nodes along the right gastroepiploic artery (no. 4d) and parapyloric nodes (nos. 5, 6)) offered negligible therapeutic benefit. Their finding that extension of a gastrectomy procedure did not clearly improve the survival outcomes of Siewert type II patients might be the reason why the outcomes between PG and TG are comparable.

Although the current AJCC staging manual for gastric cancer requires that an assessment of retrieved lymph nodes is ≥15 examined lymph nodes for therapy and staging [22], the UICC staging system and other research recommend 12 examined lymph nodes as the optimal baseline for a lymphadenectomy to obtain long-term survival benefits [4, 23]. In our present cohort, although the number of regional lymph nodes in the TG group (median, 17) was greater than that of the PG group (median, 15), the number of lymph nodes retrieved in the PG group is adequate for therapy and staging.

However, in order to achieve a microscopically curative (R0) resection, a total gastrectomy tends to be performed for cases with a larger tumor or deeper tumor invasion. The difference in the median tumor sizes of our PG group (35 mm) and TG group (40 mm) was significant (*p* < 0.001). TG has been described as a routine procedure that is appropriate for advanced adenocarcinoma and PG as suitable only for some early carcinomas in AEG [9]. However, the multivariate Cox proportional hazards model we used herein revealed that the T stage (T1–3) and the tumor size were not independent predictors of survival, and there was no significant difference in the AJCC stage between the PG and TG approaches in our subgroup analysis. It appears that as long as the tumor is completely removed, the tumor size and the depth of invasion do not affect the prognosis of the patient regardless of the tumor stage, but a larger tumor size coincides with a longer time of progression and is associated with local lymph node metastasis.

The N stage has been regarded as a powerful determinant of the clinical outcomes in Siewert type II AEG, and this was demonstrated in an earlier study [24]. In the present study's 6th AJCC stage subgroup analysis, we observed no difference between PG and TG in stage I to stage III. Therefore, in both the early and locally advanced AEG cases, PG was equivalent to TG in survival outcomes [21].

Intriguingly, our subgroup analyses revealed that older patients (age ≥ 65 years) who underwent a PG achieved better long-term overall survival compared to those who underwent a TG. We identified 70 years as the optimal age to render the maximal distinction between a PG group and a TG group. Although the exact reasons for the survival disadvantage of older AEG patients who undergo a TG remain unknown, the rates of concomitant diseases and comorbidities of the elderly are relatively greater than those of younger patients, increasing the risk in the TG procedure. In addition, the preservation of an adequate remnant of the distal stomach in PG surgery reserves some of the body's physiological function, and this may contribute to the survival results.

Although we examined a large patient sample with normative follow-up information in the SEER database, there were still several study limitations; i.e., the lack of information on comorbidities, nutrition status, and the quality of surgery, all of which may influence the short- and long-term survival of the patients (especially among the elderly patients). Another limitation is the lack of information about the surgical margin status used to confirm residual tumor, which powerfully influences patients' long-term survival [22]. Biases associated with unmeasured parameters might also arise, because the information regarding the administration of chemotherapeutic or radiotherapeutic agents to our cohort was absent; only the choice of “yes” versus “no or unknown” was available. Despite this limitation, the SEER registry remains a valuable database for analyzing the epidemiology, survival outcomes, and cancer therapies of patients.

Finally, in this retrospective cohort study, there was no analysis of extended esophagectomy as a third surgical procedure. We intentionally chose to omit the procedure of extended esophagectomy because the report of a study including a SEER database analysis had clearly shown its equality with total gastrectomy [25].

In conclusion, there was an equivalent survival benefit between PG and TG for both early-stage and locally advanced-stage AEG, but proximal gastrectomy could be considered the optimal approach for elderly patients with Siewert type II AEG.

## Figures and Tables

**Table 1:** Characteristics among AEG patients before and after PSM.

Characteristics	Raw data					Adjusted data				
	PG		TG		*p*	PG		TG		*p*
	(*N* = 1584)		(*N* = 633)			(*N* = 627)		(*N* = 627)		
Age					0.532					0.572
<65 years	800	50.5%	329	52.0%		314	50.1%	324	51.7%	
≥65 years	784	49.5%	304	48.0%		313	49.9%	303	48.3%	
Period					0.471					0.955
2004-2009	885	55.9%	343	54.2%		340	54.2%	341	54.4%	
2010-2014	699	44.1%	290	45.8%		287	45.8%	286	45.6%	
Gender					0.285					0.171
Male	1290	81.4%	503	79.5%		518	82.6%	499	79.6%	
Female	294	18.6%	130	20.5%		109	17.4%	128	20.4%	
Race					<0.01					0.929
White	1427	90.1%	510	80.6%		515	82.1%	510	81.3%	
Black	45	2.8%	49	7.7%		42	6.7%	43	6.9%	
Others	112	7.1%	74	11.7%		70	11.2%	74	11.8%	
Marital status					0.954					0.707
Married	1123	70.9%	448	70.8%		453	72.2%	447	71.3%	
Others	461	29.1%	185	29.2%		174	27.8%	180	28.7%	
Grade					0.155					0.838
Well/moderately	688	43.4%	254	40.1%		261	41.6%	252	40.2%	
Poorly/undifferentiated	896	56.6%	379	59.9%		366	58.4%	375	59.8%	
AJCC stage					0.411					0.959
Stage IA	301	19.0%	104	16.4%		104	16.6%	104	16.6%	
Stage IB	326	20.6%	128	20.2%		121	19.3%	128	20.4%	
Stage II	545	34.4%	233	36.8%		238	38.0%	229	36.5%	
Stage IIIA	346	21.8%	134	21.2%		134	21.4%	132	21.1%	
Stage IIIB	66	4.2%	34	5.4%		30	4.8%	34	5.4%	
AJCC T stage					0.028					0.997
T1	390	24.6%	131	20.7%		131	20.9%	131	20.9%	
T2	816	51.5%	365	57.7%		360	57.4%	359	57.3%	
T3	378	23.9%	137	21.6%		136	21.7%	137	21.9%	
AJCC N stage					0.162					0.901
N0	633	40.0%	236	37.3%		231	36.8%	236	37.6%	
N1	756	47.7%	301	47.6%		305	48.6%	297	47.4%	
N2	195	12.3%	96	15.2%		91	14.5%	94	15.0%	
Tumor size (mm)					<0.01					0.959
1-20	381	24.1%	111	17.5%		112	17.9%	111	17.7%	
21-50	821	51.8%	321	50.7%		312	49.8%	317	50.6%	
>50	382	24.1%	201	31.8%		203	32.4%	199	31.7%	
Chemotherapy					0.029					0.907
No/unknown	658	41.5%	231	36.5%		229	36.5%	231	36.8%	
Yes	926	58.5%	402	63.5%		398	63.5%	396	63.2%	
Radiation					0.515					0.611
None/unknown	845	53.3%	328	51.8%		317	50.6%	326	52.0%	
Beam radiation	739	46.7%	305	48.2%		310	49.4%	301	48.0%	
Neoadjuvant radiation					0.146					0.175
None/unknown	115	72.8%	480	75.8%		455	72.5%	476	75.9%	
Yes	430	27.2%	153	24.2%		172	27.5%	151	24.1%	

**Table 2:** Univariate and multivariate analyses for 1,254 AEG patients.

Characteristics		Univariate analysis		Multivariate analysis	
		HR [95% CI]	*p*	HR [95% CI]	*p*
Age	<65 years	1	<0.01	1	<0.01
	≥65 years	1.509 [1.296-1.758]		1.650 [1.412-1.928]	
Marital status	Married	1	0.001	1	0.003
	Others	1.329 [1.126-1.568]		1.292 [1.093-1.527]	
Grade	Well/moderately	1	<0.01	1	0.017
	Poorly/undifferentiated	1.486 [1.267-1.743]		1.220 [1.036-1.436]	
AJCC stage	Stage IA	1	<0.01	1	<0.01
	Stage IB	2.391 [1.738-3.288]	<0.01	2.121 [1.528-2.942]	<0.01
	Stage II	3.146 [2.349-4.212]	<0.01	2.318 [1.576-3.408]	<0.01
	Stage IIIA	3.716 [2.734-5.051]	<0.01	2.283 [1.467-3.555]	<0.01
	Stage IIIB	3.706 [2.428-5.655]	<0.01	1.837 [1.022-3.302]	0.042
AJCC N stage	N0	1	<0.01	1	<0.01
	N1	2.022 [1.690-2.419]	<0.01	1.447 [1.096-1.911]	0.009
	N2	2.601 [2.067-3.274]	<0.01	2.429 [1.620-3.641]	<0.01
Surgery	Proximal gastrectomy	1	0.139	1	0.012
	Total gastrectomy	1.121 [0.963-1.305]		1.216 [1.043-1.418]	
Regional lymph nodes examined	1-10 nodes	1	<0.01	1	<0.01
	11-20 nodes	0.743 [0.624-0.884]	0.001	0.635 [0.532-0.759]	<0.01
	>20 nodes	0.651 [0.533-0.794]	<0.01	0.530 [0.431-0.651]	<0.01
Year	2004-2009	1	0.020	—	0.266
	2010-2014	0.815 [0.687-0.968]		—	
AJCC T stage	T1	1	<0.01	—	0.963
	T2	2.285 [1.814-2.879]	<0.01	—	0.963
	T3	2.213 [1.699-2.881]	<0.01	—	0.963
Tumor size (mm)	1-20	1	<0.01	—	0.289
	21-50	1.713 [1.341-2.188]	<0.01	—	0.679
	>50	2.171 [1.684-2.800]	<0.01	—	0.205
Gender	Male	1	0.726		
	Female	0.815 [0.687-0.968]			
Race	White	1	0.464		
	Black	1.021 [0.751-1.388]	0.896		
	Others	0.858 [0.670-1.098]	0.224		
Chemotherapy	No/unknown	1	0.632		
	Yes	0.963 [0.823-1.125]			
Radiation	None/unknown	1	0.584		
	Beam radiation	0.958 [0.823-1.116]			
Neoadjuvant radiation	None/unknown	1	0.875		
	Yes	0.989 [0.862-1.135]			

## Data Availability

The data used to support the findings of this study are available from the corresponding authors upon request.
